# The Role of KEAP1-NRF2 System in Atopic Dermatitis and Psoriasis

**DOI:** 10.3390/antiox11071397

**Published:** 2022-07-19

**Authors:** Tatsuya Ogawa, Yosuke Ishitsuka

**Affiliations:** 1 Department of Dermatology, Faculty of Medicine, University of Tsukuba, 1-1-1 Tennodai, Tsukuba 305-8575, Ibaraki, Japan; ishitsuka@derma.med.osaka-u.ac.jp; 2 Department of Dermatology, Osaka University Graduate School of Medicine, 2-2 Yamadaoka, Suita 565-0871, Osaka, Japan

**Keywords:** antioxidant, atopic dermatitis, dimethyl fumarate, eczema, KEAP1-NRF2 system, mouse model, psoriasis, redox

## Abstract

The Kelch-like erythroid cell-derived protein with cap‘n’collar homology-associated protein 1 (KEAP1)-nuclear factor erythroid-2-related factor 2 (NRF2) system, a thiol-based sensor-effector apparatus, exerts antioxidative and anti-inflammatory effects and maintains skin homeostasis. Thus, NRF2 activation appears to be a promising treatment option for various skin diseases. However, NRF2-mediated defense responses may deteriorate skin inflammation in a context-dependent manner. Atopic dermatitis (AD) and psoriasis are two common chronic inflammatory skin diseases caused by a defective skin barrier, dysregulated immune responses, genetic predispositions, and environmental factors. This review focuses on the role of the KEAP1-NRF2 system in the pathophysiology of AD and psoriasis and the therapeutic approaches that utilize this system.

## 1. Introduction

The epidermis is the interface between an organism and its external environment. The stratum corneum (SC), the outermost layer of the epidermis, prevents dehydration and invasion of external pathogens and allergens. The Kelch-like erythroid cell-derived protein with cap‘n’collar homology-associated protein 1 (KEAP1)-nuclear factor erythroid-2-related factor 2 (NRF2) system, a thiol-based sensor-effector apparatus, maintains skin reduction-oxidation (redox) balance [1]. KEAP1 senses oxidative damage through reactive cysteine residues and activates NRF2-mediated antioxidative responses in keratinocytes (KCs) [2]. A previous study showed that *Keap1*-knockout mice display constitutive nuclear accumulation of NRF2 and exhibit weaning-age lethality, due to orthohyperkeratosis of the esophagus and forestomach, resulting in malnutrition [3]. To date, there has been an increasing amount of evidence that underscores the indispensable functions of the KEAP1-NRF2 system in epidermal keratinization and various skin disorders [4,5]. These studies suggest that NRF2 plays a key role in skin homeostasis [2].

Atopic dermatitis (AD) and psoriasis are two common chronic recurrent inflammatory skin disorders that affect a substantial number of patients worldwide and can compromise their quality of life. Both AD and psoriasis are characterized by a defective skin barrier, dysregulated immune responses (i.e., type 2 immunity or interleukin [IL]-4/IL-13 axis inflammation in AD and type 3 immunity or IL-23/IL-17 axis inflammation in psoriasis), genetic predispositions, and causative environmental factors [6,7]. Moreover, we have recently shown that impaired redox homeostasis in the epidermis may play a crucial role in the pathogenesis of AD and psoriasis [8,9].

Here, we review the significant roles of the KEAP1-NRF2 system in the pathophysiology of AD and psoriasis and the therapeutic approaches that utilize this system. We conducted a literature review by searching studies that examined the activation status of NRF2 in clinical samples from human AD/psoriasis, as well as experimental works in which the efficacy of therapies targeting the KEAP1-NRF2 system was analyzed in vivo/vitro. The PubMed search was performed using the terms “NRF2 and atopic dermatitis” or “NRF2 and psoriasis”.

## 2. Overview of the KEAP1-NRF2 System

The cytoplasmic protein KEAP1 acts as a sensor for oxidative insults, and the transcription factor NRF2 serves as an effector for counter responses [1]. Under normal conditions, NRF2 is constantly polyubiquitinated by the KEAP1-CULIN3 (CUL3) ubiquitin E3 ligase complex and is subjected to rapid proteasome-mediated degradation in the cytoplasm. However, when cells are exposed to electrophiles or reactive oxygen species (ROS), the reactive cysteine residues of KEAP1 are covalently modified and KEAP1-CUL3 ubiquitin E3 ligase activity decreases. As a result, NRF2 accumulates and translocates to the nucleus, dimerizes with the small Maf protein, and binds to the antioxidant/electrophile-response element in the enhancer/promoter region of target genes. The actin-associated KEAP1 protein localizes to the perinuclear region of the cytoplasm, and thus acts as a “floodgate” for the nuclear entry of NRF2 [1]. NRF2 activates a battery of cytoprotective genes that are involved in glutathione (GSH) synthesis (glutamate-cysteine ligase catalytic subunit and glutamate-cysteine ligase modifier subunit), ROS elimination (thioredoxin reductase 1 and peroxiredoxin 1), phase II detoxification (GSH S-transferase and NAD(P)H quinone dehydrogenase 1 [NQO1]), and drug excretion (multidrug resistance-associated protein 1) [1].

## 3. NRF2 as a Critical Regulator of Immune Responses

NRF2 exerts robust anti-inflammatory activity by inducing antioxidant response genes, quenching ROS, and promoting cell survival [1]. Experimental studies using *Nrf2*-knockout mice have suggested that NRF2 negatively regulates both innate and adaptive arms of immune responses. *Nrf2*-knockout mice exhibited exacerbation of lipopolysaccharide (LPS)-induced sepsis-like inflammation, which was alleviated by treatment with NRF2 activators, such as CDDO-imidazolide [10] or a cysteine antioxidant *N*-acetylcysteine (NAC) [11]. These results clearly indicate that NRF2 negatively controls innate immunity. Moreover, NRF2 has been shown to regulate autoimmune inflammation. *Nrf2*-knockout mice displayed an exacerbation of rheumatoid arthritis [12] and systemic lupus erythematosus [13,14]. Correspondingly, systemic activation of NRF2 by *Keap1* knockdown suppressed effector T cell activities in the scurfy mice, which exhibit lethal multiorgan inflammation, owing to the lack of functional regulatory T cells (Tregs) [15].

## 4. NRF2 as a Driver of Tissue-Repairing Inflammation in the Skin

Type 2 immunity, in which IL-4 and IL-13 are the signature cytokines, functions to protect the skin against metazoan parasites and mediates host protection through tissue repair and inflammation control [16]. Type 2 immunity consists of group 2 innate lymphoid cells (ILC2s), CD8^+^ cytotoxic T (Tc2) cells, and CD4^+^ helper T (Th2) cells, which activate mast cells, basophils, and eosinophils, and promote immunoglobulin E (IgE) production [16]. Conversely, excessive type 2 immunity may cause allergies (e.g., AD or asthma) or fibrosis [17]. Physical injuries can promote the release of damage-associated molecular patterns (DAMPs) and induce type 2 immunity [17]. DAMPs represent a heterogeneous group of molecules that originate from extracellular or intracellular spaces [18]. For example, uric acid crystals [19] or extracellular adenosine triphosphate (ATP) [20] that are released from damaged tissues can drive type 2 immunity in the respiratory system. IL-1α is a ubiquitous pro-inflammatory cytokine stored as a bioactive precursor in almost all cell types, including KCs [21]. Unlike IL-1β, whose bioactivity is strictly regulated by the inflammasome, both the precursor and cleaved form of IL-1α possess biological activity [21]. This unique property makes IL-1α a critical upstream inflammatory cue [21] that is released from tissues with injuries, such as cellular deformation [22]. In the epidermis, IL-1α promotes the recovery of the barrier function by promoting the synthesis and secretion of lipids and antimicrobial peptides [23,24]. Because NRF2 is a critical regulator of epidermal barrier function, as discussed later, KCs from *Nrf2*-knockout mice released significantly smaller amounts of IL-1α than that in wild-type KCs, and *KEAP1* silencing in cultured normal human epidermal keratinocytes (NHEK) increased *IL1A* mRNA expression [8]. Therefore, NRF2 appears to regulate intracellular IL-1α expression levels in KCs. Similarly, NRF2 activation by *tert*-butylhydroquinone (TBHQ) in T cells skewed CD4^+^ T cells toward Th2 differentiation [25]. Collectively, NRF2 may promote IL-1α–type 2 immunity-mediated inflammatory responses to maintain skin homeostasis.

Type 3 immunity, which is characterized by effector cytokines IL-17 and IL-22, primarily protects the skin against extracellular bacteria and fungi and plays a key role in the initiation and maintenance of several autoimmune diseases, including psoriasis and multiple sclerosis (MS) [16]. Effector cells in type 3 immunity consist of ILC3s, Tc17 cells, and Th17 cells, which recruit mononuclear phagocytes and neutrophils to tissues and induce epithelial antimicrobial responses [16]. The pro-inflammatory cytokines IL-1β and IL-23 drive Th17 cell differentiation [16]. Before becoming biologically active, IL-1β undergoes nucleotide-binding oligomerization domain-like receptor pyrin domain-containing 3 (NLRP3) inflammasome-mediated cleavage [26]. NRF2 activators, such as TBHQ, sulforaphane, and xanthohumol, suppress NLRP3 inflammasome activation [27]. Moreover, in a study on experimental autoimmune uveitis, sodium butyrate suppressed Th17 cell differentiation but reciprocally induced Treg differentiation through the activation of the NRF2/heme oxygenase-1 pathway [28]. Therefore, NRF2 appears to dampen the IL-1β–type 3 immunity-mediated inflammatory responses. Given that type 2 immunity generally prevents type 1 or type 3 immunity-mediated immune pathologies [16], the KEAP1-NRF2 system may promote tissue-repair-related host-protective responses mediated by the IL-1α–type 2 axis, upon sensing superficial epithelial damage [3,8].

## 5. NRF2 as a Regulator of Keratinization

The epidermal tissue establishes a gradient of thiols, and free thiol (–SH) is effectively converted into disulfide (–S–S–) during terminal differentiation [29,30]. The redox status of protein thiol (–SH) in the differentiating layers appears to be central to the functionality of the protective barrier. For instance, SC is abundant in disulfide (–S–S–) cross-linkages, endowing the skin with mechanical resilience, as well as impermeability against pathogens and allergens [31]. The unique properties of SCs are attributed to the formation of cornified envelopes (CEs) [32], which replace the KC plasma cell membrane during the specialized cell death program termed cornification [33]. Loricrin (LOR), a thiol (–SH)-rich major CE protein [34], stabilizes the SC by inter/intra-molecular disulfide (–S–S–) cross-linkages and promotes structural maturation of the epidermal tissue [34]. Among the myriad xenobiotic metabolisms that govern epidermal homeostasis, the thiol-based sensor-effector apparatus KEAP1-NRF2 system appears to play a central role.

NRF2 maintains the thiol gradient (–SH) in the epidermis [35], but its unrestrained activation can be detrimental. Systemic or epidermis-specific constitutive NRF2 activation is achieved through germline deletion of *Keap1* [3] and transgenic introduction of the constitutively active (ca) Nrf2 gene via the keratin 5 (K5) or K10 promoter [36], respectively. These mouse models exhibited hyperkeratotic phenotypes that somewhat resembled autosomal-recessive congenital ichthyosis [3,36] or metabolizing acquired dioxin-induced skin hamartomas [37]. 

Several studies have demonstrated that NRF2 plays a crucial role in skin homeostasis. NRF2 ameliorated the ultraviolet (UV) response in the skin. Irradiation of *Nrf2*-knockout mice with UVB induced stronger and longer-lasting sunburn reactions with greater KCs apoptosis and oxidative damage than in control mice [38]. Similarly, transgenic mice expressing a caNrf2 mutant in KCs (K5cre- or K10caNrf2) showed attenuated UVB-induced KCs apoptosis and enhanced ROS detoxification [35]. NRF2 also protected the skin against the chemical carcinogen 7,12-dimethylbenz(a)anthracene (DMBA) [39,40]. Furthermore, we have recently found that LOR and the KEAP1-NRF2 system coordinately upregulate robust antioxidative responses and exert xenobiotic metabolism in the epidermis [4,5,41]. In the absence of LOR, NRF2 directly upregulated thiol-rich CE proteins, small proline-rich protein 2 (SPRR2) [42], and late cornified envelope proteins [43], thereby compensating for the disrupted thiol gradient and epidermal barrier functions. Upon exposure to DMBA, *LOR*-knockout mice exhibited increased expression levels of NRF2 and DNA damage marker phospho-histone H2A.X, which were rescued by an oral administration of NAC [44]. These results suggest that thiol-rich LOR is indispensable for the antioxidative protection of the epidermis. Furthermore, NRF2-mediated adaptive responses evoked lamellar granule secretory functions and increased the expression of corneodesmosin, an extracellular component of corneodesmosomes [45], in *LOR*-knockout mice [46]. Therefore, NRF2 confers specialized antioxidative cytoprotection to the epidermis in coordination with LOR [2,37,39,47,48,49].

Overall, NRF2 has beneficial effects on the epidermis under stress conditions (e.g., UV irradiation, chemical carcinogen exposure, and disrupted skin barriers). However, since *Keap1*-knockout mice and K5cre- or K10caNrf2 transgenic mice exhibited pathogenic hyperkeratosis, long-term unrestricted activation of NRF2 may have negative consequences [2].

## 6. Therapeutic Application of NRF2 Activators

NRF2 is a promising therapeutic target for oxidative stress-related diseases, such as autoimmune, respiratory, digestive, cardiovascular, metabolic, and neurodegenerative diseases, and cancer [50]. NRF2 activators or KEAP1 inhibitors include electrophiles, protein–protein interaction (PPI) inhibitors, and multi-target drugs (e.g., glycogen synthase kinase 3 inhibitors, HRD1 inhibitors, p62 activators, broad-complexes, tramtrack, bric-a-brac domains, and CNC homolog 1 inhibitors) [51]. Most pharmacological NRF2 activators are electrophilic molecules [52], which include bardoxolone methyl (CDDO-Me) and its derivative (RTA-408), dimethyl fumarate (DMF), monomethyl fumarate (MMF) derivative (ALKS-8700), and sulforaphane and its derivatives (SFX-01 and ITH12674) [51]. Other examples of electrophiles are TBHQ, diethyl maleate, TFM-735, and nitric oxide, although most of these compounds are still far from being used in a clinical setting [51]. TBHQ, a well-recognized electrophilic NRF2 activator, is widely used as a preservative in food because it prevents the rancidification of lipids [53]. Meanwhile, there has been an increasing interest in non-electrophilic modulators, such as PPI inhibitors, which prevent the docking of NRF2 to KEAP1 and may be more selective than electrophilic NRF2 activators [54]. However, NRF2 has controversial roles in cancer [55]; that is, NRF2 may be protective in the early stages of cancer but may be tumorigenic in the advanced stages (i.e., activated NRF2 may not only prevent ROS-induced oncogenic mutations, but also promote tumor cell survival by inhibiting apoptosis) [39]. Thus, the clinical application of NRF2 inhibitors has not been fully investigated.

## 7. NRF2 and Atopic Dermatitis

AD, also known as eczema or atopic eczema, is a common inflammatory skin disorder characterized by eczematous eruptions and intense itching [6]. AD affects over 20% of children [56] and 2.1–4.9% of adults [57] in industrialized countries. A family history of AD is associated with the development of AD [58], and filaggrin (FLG) mutations are associated with the strongest genetic risk for AD [59]. The pathogenesis of AD includes the interplay among skin barrier disruption, type 2 immunity, and pruritus [60], which can be targeted by innovative biological and small-molecule therapies. The emergence of dupilumab, an inhibitor of IL-4 and IL-13 signaling, has embodied this concept of “trinity” [61]. Topical and oral Janus kinase (JAK) inhibitors, such as delgocitinib, baricitinib, upadacitinib, and abrocitinib, have become promising treatment options for AD because they can block a battery of cytokines, growth factors, and hormone receptor signaling pathways that modulate the pathogenesis of AD [6]. AD can be accompanied by subsequent extracutaneous allergic diseases, such as asthma and allergic rhinitis [62]; therefore, early intervention with moisturizers for epicutaneous sensitization should be performed to prevent allergic march or atopic march [63].

Studies using mouse models of AD (e.g., epicutaneous application of sensitizers) [64] and analyses of human AD samples have revealed that NRF2 ameliorates AD inflammation (Table 1).

As a therapeutic target, NRF2 activation by natural compounds or chemical agents can improve the inflammatory signal of human KCs in vitro or the phenotype of sensitizer-induced skin inflammation in mouse models that mimic human AD. The details are discussed in the following sections.

### 7.1. NRF2 Activators for AD

Coal tar, which consists of a variety of polycyclic aromatic hydrocarbons (PAHs) [65], has been used to treat skin diseases for more than 2000 years [66]. Coal tar induced aryl hydrocarbon receptor (AHR)-mediated NRF2 activation and epidermal differentiation and inhibited the IL-4/ signal transducer and activator of transcription (STAT) 6 signaling pathway, thereby improving AD-like inflammation in an organotypic skin model with primary KCs obtained from AD patients [66]. Igalan, a sesquiterpene lactone from *Inula helenium* (L.), suppressed the JAK/STAT3 signaling pathway and induced KC differentiation, thereby improving inflammatory cytokine (tumor necrosis factor-alpha (TNF-α) and interferon-gamma (IFN-γ) or IL-4)-induced AD-like HaCaT cells [67].

Topical application of NRF2 activators is effective in hapten (e.g., 2,4-dinitrochlorobenzene (DNCB) or oxazolone-induced mouse models of AD. Sulforaphane, a naturally occurring isothiocyanate derived from cruciferous vegetables, such as broccoli, brussel sprouts, and cabbage, strongly induced phase II enzymes [68]. Sulforaphane suppressed the JAK1/STAT3 signaling pathway [69] and inhibited apoptosis [70] in mouse AD skin. Macakurzin C-derivative, which is isolated from the leaves of *Macaranga kurzii*, attenuated the nuclear factor kappa B (NF-κB) signaling pathway in HaCaT cells stimulated by IFN-γ and TNF-α [71]. A naturally occurring flavone chrysin derivative inhibited the NF-κB and JAK2/STAT1 signaling pathways in LPS-activated macrophages [72]. Cardamonin, a natural compound abundantly found in cardamom species, inhibited Th2 cytokines production in mouse AD skin [73].

Systemic application of NRF2 activators is also effective in mouse models of AD induced by DNCB, house dust mites (HDM), or trimellitic anhydride. The food and traditional oriental medicine *Platycodon grandiflorum* root-derived saponins (Changkil saponins) and its component platycodin D suppressed the NF-κB/STAT1 signaling pathway in HaCaT cells stimulated by TNF-α and IFN-γ [74]. 6-shogaol, a pungent compound isolated from ginger, inhibited ROS generation and mitogen-activated protein kinase signaling pathways in HaCaT cells and NHEK stimulated by TNF-α and IFN-γ [75]. *Soshiho-tang* [76] and *Chijabyukpi-tang* [77], traditional herbal medicines, inhibited pro-inflammatory cytokines and chemokines in mouse AD skin. The flavonoid quercetin, which is found in most edible fruits and vegetables, modulated the high-mobility group Box 1/receptor for advanced glycation end products/NF-κB signaling pathway in mouse AD skin [78]. Miquelianin, an active compound in *Rosae multiflorae* fructus, suppressed the proliferation of cultured CD4^+^ T cells isolated from splenocytes [79]. Collectively, NRF2 activators exert antioxidative and anti-inflammatory effects and ameliorate AD-like skin manifestations.

### 7.2. NRF2-Mediated Antioxidative Responses in the Epidermis and AD

In the lesional epidermis of human AD, however, NRF2 and its downstream target SPRR2 expression were elevated compared to the normal control epidermis, as shown by immunohistochemistry (IHC) analysis [8]. These findings were consistent with skin specimens from congenital skin disorders, such as Netherton syndrome (OMIM number 256,500) [80] and peeling skin syndrome (OMIM number 609,796) [81], which share similar features with AD [8]. Correspondingly, common environmental allergens, such as ovalbumin, HDM, and cedar pollen, stabilized NRF2 in cultured NHEKs, suggesting that skin sensitization would accompany NRF2 activation in the viable epidermal layers, which presumably takes place upon the disruption of the SC [8]. In contrast, in the peripheral blood of AD patients, protein expression level of NRF2 was downregulated compared to those in normal control patients. Kim et al. suggested that NRF2 downregulation enhances AD-associated inflammatory responses [82].

Consistent with these observations in human AD epidermis, we recently found that the immunogenicity of a hapten depends directly on NRF2-mediated antioxidant host defenses in the epidermis [8]. The specific chemical properties of haptens determine the nature of the immunological memory response. For example, immunogenic haptens, such as 1-fluoro-2,4-dinitrobenzene (DNFB) or DNCB, disrupted the reactive thiol layer in the SC, leading to dinitrophenylation and GSH depletion in the epidermis [83]. In contrast, tolerogenic haptens, such as 2,4-dinitrothiocyanobenzene (DNTB), induced dinitrophenylation exclusively in the SC [83] and preferentially led to peripheral tolerance through the generation of Treg memory [84]. Intriguingly, *Nrf2* deficiency abrogated hapten sensitization and subsequent immune responses in a hapten-induced mouse model of AD [8]. In this setting, *Nrf2* deficiency attenuated the immediate-type contact hypersensitivity response, inflammatory cell (e.g., CD4^+^ cell, eosinophil, basophil, and mast cell) infiltration, type 2 inflammatory cytokine (e.g., *Il4* and *Il13*) mRNA expression levels, and serum IgE levels [8]. In short, as opposed to the generally accepted anti-inflammatory effect of the KEAP1/NRF2 system, NRF2 can augment cutaneous tissue responses skewed toward a type 2 immunity [8]. Remarkably, *Nrf2* deficiency decreased CD4^+^ and CD8^+^ T cell proliferation and Treg induction in draining lymph nodes after a single DNFB administration but not DNTB [8]. Taken together, NRF2 may profoundly affect the initiation of tissue-protective inflammatory responses.

It appears that proper activation of NRF2 can ameliorate AD-like skin manifestations and type 2 immunity through its antioxidative and anti-inflammatory effects. In contrast, KEAP1-sensing of epidermal damage can augment innate immune responses and facilitate skin sensitization as a result of increased expression levels of DAMPs, such as IL-1α [8].

**Table 1:** NRF2 and atopic dermatitis.

Species	Administration Route	Cell Type	Treatment	NRF2 Status	Effect of Treatment	Reference
Human	N/A	AD skin	N/A	Upregulation in skin	N/A	[8]
Human	N/A	Peripheral blood of AD	N/A	Downregulation in blood	N/A	[82]
Human	In vitro	Organotypic skin models with primary KCs from AD patients	Coal tar	Upregulation in KCs	Induction of epidermal differentiation Inhibition of Th2 cytokine signaling	[66]
Human	In vitro	TNF-α and IFN-γ or IL-4-induced AD-like HaCaT cells	Igalan	Upregulation in HaCaT cells	Inhibition of JAK/STAT3	[67]
Mouse	N/A	TNCB-induced AD-like skin	Gene knockout of *Nrf2*	Systemic downregulation	Amelioration of AD-like skin inflammation	[8]
Mouse	Topical	DNCB-induced AD-like skin	Sulforaphane	Upregulation in skin	Inhibition of JAK1/STAT3 Amelioration of AD-like skin inflammation	[69]
Mouse	Topical	OX-induced AD like skin	CPD 14	Upregulation in HaCaT cells	Inhibition of NF-κB Amelioration of AD-like skin inflammation	[71]
Mouse	Topical	OX-induced AD like skin	CPD 6	Upregulation in macrophages	Inhibition of NF-κB and JAK2/STAT1 Amelioration of AD-like skin inflammation	[72]
Mouse	Topical	OX-induced AD like skin	Cardamonin	Upregulation in skin	Downregulation of Th2 cytokines Amelioration of AD-like skin inflammation	[73]
Mouse	Subcutaneous	DNCB-induced AD-like skin	Sulforaphane	Upregulation in skin	Inhibition of apoptosis in skin Amelioration of AD-like skin inflammation	[70]
Mouse	Oral	DNCB-induced AD-like skin	CKS Platycodin D	Upregulation in HaCaT cells	Inhibition of NF-κB/STAT1 Amelioration of AD-like skin inflammation	[74]
Mouse	Oral	DNCB-induced AD-like skin	6-shogaol	Upregulation in skin	Inhibition of ROS and MAPKs Amelioration of AD-like skin inflammation	[75]
Mouse	Oral	DNCB-induced AD-like skin	SST	Upregulation in skin	Downregulation of pro-inflammatory cytokines and chemokines Amelioration of AD-like skin inflammation	[76]
Mouse	Oral	DNCB-induced AD-like skin	CBT	Upregulation in skin	Downregulation of pro-inflammatory cytokines and chemokines Amelioration of AD-like skin inflammation	[77]
Mouse	Oral	HDM-induced AD-like skin in NC/Nga transgenic mouse	Quercetin	Upregulation in skin	Inhibition of HMGB1/RAGE/NF-κB Amelioration of AD-like skin inflammation	[78]
Mouse	Oral	TMA-induced AD like skin	MQL	Upregulation in CD4^+^ T cells	Inhibition of CD4^+^ T cells proliferation Amelioration of AD-like skin inflammation	[79]

N/A, not applicable; AD, atopic dermatitis; CBT, *Chijabyukpi-tang*; CKS, Changkil saponins; CPD 6, chrysin-derivative; CPD 14, macakurzin C-derivative; DNCB, 2,4-dinitrochlorobenzene; HDM, house dust mite; HMGB1, high-mobility group box 1; IFN-γ, interferon-gamma; IL-4, interleukin-4; JAK, Janus kinase; KC, keratinocyte; MAPK, mitogen-activated protein kinase; MQL, miquelianin; NF-κB, nuclear factor kappa B; OX, oxazolone; RAGE, receptor for advanced glycation end products; SST, *Soshiho-tang*; STAT1/3, signal transducer and activator of transcription 1/3; Th2, type 2 helper T; TMA, trimellitic anhydride; TNCB, 2,4,6-trinitro-1-chlorobenzene; TNF-α, tumor necrosis factor-alpha.

## 8. NRF2 and Psoriasis

Psoriasis is a chronic recurrent inflammatory skin disease characterized by aberrant hyperproliferation and differentiation of KCs, and immune cell infiltration in the dermis and epidermis [7]. Approximately 125 million people suffer from psoriasis worldwide [85]. Psoriasis has a strong genetic predisposition, and the human leukocyte antigen (HLA)-class 1 allele HLA-C*06:02 is the main risk psoriasis gene [86]. In the pathogenesis of psoriasis, IL-23-mediated activation of the Th17 pathway is regarded as the central inflammatory cascade [87], and biologics, such as TNF-α inhibitors, IL-23 inhibitors, and IL-17 inhibitors, have been used for the treatment of psoriasis [85]. Severe psoriasis can be accompanied by systemic inflammation, which eventually cause cardiovascular comorbidity, namely “psoriatic march” [88]. ROS-mediated oxidative stress is also closely associated with the pathogenesis of psoriasis [89]; therefore, electrophilic agents, such as fumaric acid esters (FAEs), can evoke antioxidative responses and improve psoriasis inflammation [90]. FAEs were first investigated for psoriasis treatment in 1959 [91]. Fumaderm^®^ (Biogen Inc., Cambridge, MA, USA) is a mixture of DMF and MMF salts. In 1994, Fumaderm^®^ was approved as a systemic treatment for severe psoriasis, and in 2008, as a systemic treatment for moderate-to-severe psoriasis in Germany [92]. Skilarence^®^ (Almirall, S.A., Barcelona, Spain) is a novel oral formulation of DMF. In 2017, Skilarence^®^ was approved by the European Medicines Agency for the treatment of moderate-to-severe plaque psoriasis in Europe [92]. The main clinical effect of FAEs is immunomodulation, wherein NF-κB-mediated inflammatory cascades are inhibited [92]. Of note, FAEs exert neuroprotective effects via NRF2 activation in a mouse model of chronic MS (experimental autoimmune encephalomyelitis) [93]. Tecfidera^®^ (Biogen Inc.) was approved by the Food and Drug Administration and Pharmaceuticals and Medical Devices Agency for relapsing-remitting MS [94], also known as Th17-mediated autoimmune demyelinating disease. Therefore, the therapeutic efficacy of FAEs largely depends on the activation of the KEAP1/NRF2 system.

Similar to AD, cumulative evidence has shown that NRF2 can both alleviate and exacerbate psoriasiform dermatitis (Table 2). Natural compounds or chemical agents that activate NRF2 can ameliorate mouse models of psoriasis. Examples of these agents are described below.

### 8.1. NRF2-Mediated Antioxidative Responses in the Psoriatic Tissues

Previous studies have revealed that expression level of NRF2 in lesional psoriatic skin compared to those in normal control skin are inconsistent between IHC or real-time quantitative polymerase chain reaction analyses [95,96,97]. Lesional psoriatic skin showed lower mRNA expression levels of *NRF2* and its downstream targets *NQO1*, *LOR*, and *FLG* than that in non-lesional (perilesional) psoriatic skin [9]. Correspondingly, *KEAP1* mRNA expression was decreased in lesional psoriatic skin, compared to that of non-lesional (perilesional) psoriatic skin [98]. Considering that the epidermal thiol gradient is disrupted in psoriatic lesions, it would be natural to assume that the KEAP1-NRF2 system per se is abrogated by psoriasiform tissue reactions. NRF2 has been shown to be upregulated in other experiments examining granulocytes [99] and lymphocytes [100] in peripheral blood mononuclear cells obtained from patients with psoriasis and fibroblasts [101] in psoriatic skin samples. However, it should be noted that blood cells may not reflect the local redox status, and skin biopsy specimens may include both lesional and non-lesional (perilesional) psoriatic skin.

Mouse models of psoriatic skin inflammation that mimic human psoriasis have shown that NRF2 can either be beneficial or detrimental to psoriasis. We have shown that, in an imiquimod (IMQ)-induced mouse model of psoriasis [102], *Nrf2* deficiency exacerbated psoriatic skin inflammation and aberrant keratinization by upregulating mRNA expression levels of inflammatory cytokines (i.e., *Il6*, *Tnf*, *Il23a*, and *Il17a*) and protein expression level of phosphorylated STAT3, and downregulating protein expression levels of epidermal differentiation markers (i.e., K10, FLG, and LOR) [9]. Remarkably, *Nrf2* deficiency caused prominent hypogranulosis and parakeratosis [9], the hallmarks of the psoriatic tissue reaction [103]. In contrast to our results, *Nrf2* small interfering RNA (siRNA) intervention ameliorated epidermal hyperplasia and reduced protein and mRNA expression levels of stress-induced keratin (i.e., K6, K16, and K17) in this murine model [97]. Yang et al. proposed that NRF2 activation in KCs by inflammatory cytokines (e.g., IL-17 and IL-22) promotes KC proliferation through the upregulation of K6/K16/K17 and releases inflammatory cytokines and chemokines [97]. The actin-related protein 2/3 (Arp2/3) complex, which assembles branched actin filaments, inhibited NRF2 activity and influences epidermal morphogenesis and homeostasis [104]. Consequently, Arp2/3 complex subunit 4 (*Arpc4*)-knockout mice showed upregulation of NRF2 and developed spontaneous severe psoriasis-like skin inflammation with hyperkeratosis, parakeratosis, and acanthosis [104]. Consistent with this, human psoriatic skin and IMQ-induced psoriatic skin showed decreased ARPC4 protein expression levels through IHC analysis [104]. Van der Kammen et al. suggested that the depletion of the Arp2/3 complex enables NRF2 to enhance the transcription of psoriasis-related genes [104], which may include K6/K16/K17. These phenotypic discrepancies among *Nrf2*-knockout, *Nrf2*-siRNA-treated, and *Arpc4*-knockout mice may be attributed to the difference in the routes of NRF2 intervention (i.e., systemic depletion of NRF2 in *Nrf2*-knockout mice and local down- or upregulation of NRF2 in *Nrf2* siRNA-treated and *Arpc4*-knockout mice).

### 8.2. NRF2 Activators for In Vitro Models of Psoriasis

The oral hypoglycemic agent metformin is commonly used for the treatment of type 2 diabetes mellitus [105]. Metformin can be beneficial in various skin diseases via its hyperinsulinemic and hyperandrogenic effects [105]. Metformin attenuated the rapidly accelerated fibrosarcoma-1-extracellular signal-regulated kinase 1/2-NRF2 signaling pathway, thereby contributing to intracellular ROS generation and apoptosis in HaCaT cells [106]. Thus, Wang et al. suggested that metformin could be an anti-psoriasis drug that reduces NRF2 expression [106].

### 8.3. Topical NRF2 Activators for Mouse Models of Psoriasis

Topical application of NRF2 activators is effective in IMQ- and 12-O-tetradecanoylphorbol-13-acetate-induced skin inflammation models that mimic human psoriasis. Tussilagonone, a compound derived from the medicinal plant *Tussilago farfara* L., inhibited activation of the NF-κB and STAT3 signaling pathways in mouse psoriatic skin [107]. Mammalian target of rapamycin (mTOR) is a protein kinase that regulates cell growth, proliferation, and survival [108]. The mTOR inhibitor rapamycin is associated with the regulation of autophagy [109] and the dysfunction of which could contribute to the pathogenesis of psoriasis [110]. Rapamycin restored suppressed autophagy and increased AHR expression by PAH, 2,3,7,8-tetrachlorodibenzo-p-dioxin, in mouse psoriasis skin [111]. Galangin, an active flavonoid extracted from *Alpinia officinarum*, *Alpina galanga*, and propolis, downregulated the NF-κB signaling pathway in mouse psoriatic skin [112]. Perillyl alcohol, an essential oil obtained from several plants, such as citrus peel, cherries, and mint, modulated the NF-κB and STAT3 signaling pathways in mouse psoriatic skin [113]. *Moringa oleifera* L., also known as horseradish tree, suppressed Th17-related cytokines (e.g., IL-23p19, IL-17A, and IL-22) in mouse psoriatic skin [114].

### 8.4. Systemic NRF2 Activators for Mouse Models of Psoriasis

Systemic application of NRF2 activators is also effective in an IMQ-induced mouse model of psoriasis. Astilbin, isolated from a commonly used herbal medicine, reduced ROS accumulation and vascular endothelial growth factor expression in mouse psoriatic skin [115]. Of note, intragastric administration of DMF not only attenuated ear swelling and mRNA expression levels of inflammatory cytokines (i.e., *Il6*, *Tnf*, *Il23a*, and *Il17a*), but also increased mRNA expression levels of epidermal differentiation markers (i.e., *Flg* and *Lor*) in an *Nrf2*-dependent manner [9]. However, topical application of DMF has not been successful in the treatment of psoriasis, owing to the occurrence of contact dermatitis [116]. This observation suggests that the administration routes of DMF may determine the consequences of the treatment. That is, epidermis-specific NRF2 activation by topical electrophiles may facilitate further skin sensitization [8], while systemic NRF2 activation by oral electrophiles may exert immune modulation [9]. Similarly, topical exposure of TBHQ can cause contact dermatitis [117], whereas TBHQ has been shown to inhibit immune responses [118,119]. Isosorbide DMF (IDMF), a prodrug of DMF, was synthesized to eliminate the skin-sensitizing side effects [120]. IDMF strongly activated NRF2 compared to DMF in vitro and topical application of IDMF ameliorated IMQ-induced psoriatic skin inflammation [120]. Conversely, gallic acid (GA) treatment showed negative effects on NRF2 activation in psoriasis. GA, a natural small molecule found in *Radix Paeoniae Rubra*, downregulated NRF2 and its targets K16 and K17 in mouse psoriatic skin [121].

Collectively, NRF2 can attenuate psoriatic skin inflammation via antioxidative and anti-inflammatory activities but may promote KC proliferation as a consequence of the tissue-protective response. Although DMF has been shown to be a clinically and experimentally effective agent for psoriasis, future applications of NRF2 activators would need more consideration.

**Table 2:** NRF2 and psoriasis.

Species	Administration Route	Cell Type	Treatment	NRF2 Status	Effect of Treatment	Reference
Human	N/A	Psoriatic skin	N/A	Upregulation in skin	N/A	[97]
Human	N/A	Psoriatic skin	N/A	Upregulation in skin	N/A	[95]
Human	N/A	Psoriatic skin	N/A	Downregulation in skin	N/A	[96]
Human	N/A	Psoriatic skin	N/A	Downregulation in skin	N/A	[9]
Human	N/A	Psoriatic granulocytes	N/A	Upregulation in granulocytes	N/A	[99]
Human	N/A	Psoriatic lymphocytes	N/A	Upregulation in lymphocytes (PsV > PsA)	Exacerbation of pro-oxidative conditionsUpregulation of pro-apoptotic pathway	[100]
Human	N/A	Psoriatic fibroblasts	N/A	Upregulation in fibroblasts	N/A	[101]
Human	In vitro	HaCaT cells	MET	Downregulation in HaCaT cells	Induction of cell apoptosis Elevation of intracellular ROS	[106]
Mouse	N/A	Mice epidermis Cultured mice KCs	Gene knockout of *Arpc4*	Upregulation in skin	Development of psoriasis-like disease	[104]
Mouse	N/A	IMQ-induced psoriatic skin	*Nrf2* siRNA	Downregulation in skin	Amelioration of psoriatic inflammation	[97]
Mouse	N/A	IMQ-induced psoriatic skin	Gene knockout of *Nrf2*	Systemic downregulation	Exacerbation of psoriatic inflammation	[9]
Mouse	Topical	IMQ-induced psoriatic skin	TGN	Upregulation in HaCaT cells	Inhibition of NF-κB and STAT3 Amelioration of psoriatic inflammation	[107]
Mouse	Topical	IMQ-induced psoriatic skin	Rapamycin	Upregulation in skin	Restoration of suppressed autophagy and increased AHR expression Amelioration of psoriatic inflammation	[111]
Mouse	Topical	IMQ-induced psoriatic skin	GAL	Upregulation in skin	Inhibition of NF-κBAmelioration of psoriatic inflammation	[112]
Mouse	Topical	IMQ-induced psoriatic skin	IDMF	Upregulation in HEK293	Amelioration of psoriatic inflammation	[120]
Mouse	Topical	IMQ-induced psoriatic skin	POH	Upregulation in skin	Inhibition of NF-κB and STAT3 Amelioration of psoriatic inflammation	[113]
Mouse	Topical	IMQ-induced psoriatic skin	GA	Downregulation in skin	Downregulation of K16 and K17 Amelioration of psoriatic inflammation	[121]
Mouse	Topical	TPA-induced psoriatic skin	*Moringa oleifera* seeds	Upregulation in skin	Downregulation of Th17-related cytokines Amelioration of psoriatic inflammation	[114]
Mouse	Intragastric	IMQ-induced psoriatic skin	Astilbin	Upregulation in HaCaT cells	Inhibition of ROS and VEGF Amelioration of psoriatic inflammation	[115]
Mouse	Intragastric	IMQ-induced psoriatic skin	DMF	Upregulation in skin	Downregulation of inflammatory cytokines Upregulation of epidermal differentiation markers Amelioration of psoriatic inflammation	[9]

N/A, not applicable; AHR, aryl hydrocarbon receptor; Arpc4, actin-related protein 2/3 complex subunit 4; DMF, dimethyl fumarate; GA, gallic acid; GAL, galangin; HEK293, human embryonic kidney 293; IDMF, isosorbide dimethyl fumarate; IMQ, imiquimod; K16/17, keratin 16/17; KC, keratinocyte; MET, metformin; NF-κB, nuclear factor kappa B; POH, perillyl alcohol; PsA, psoriatic arthritis; PsV, psoriasis vulgaris; ROS, reactive oxygen species; siRNA, small interfering RNA; STAT3, signal transducer and activator of transcription 3; TGN, tussilagonone; Th17, type 17 helper T; TPA, 12-O-tetradecanoylphorbol-13-acetate; VEGF, vascular endothelial growth factor.

## 9. Conclusions

**Figure 1:**
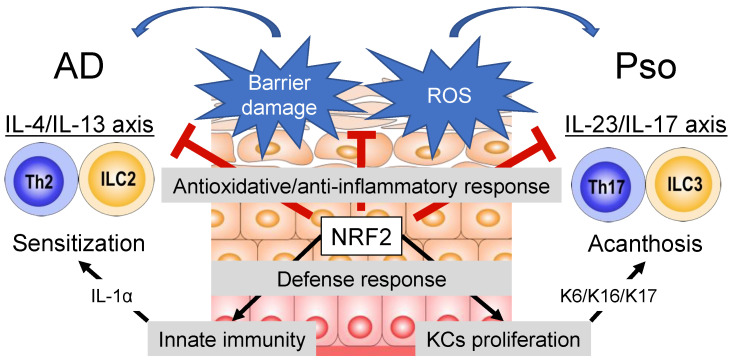
The KEAP1-NRF2 system in the pathophysiology of atopic dermatitis and psoriasis. NRF2 exerts antioxidative and anti-inflammatory effects, thereby ameliorating IL-4/IL-13 or IL-23/IL-17 axis inflammation and skin manifestations. In contrast, NRF2 can induce tissue-protective responses, which may initiate innate immunity activation or keratinocyte proliferation. AD, atopic dermatitis; IL, interleukin; ILC, innate lymphoid cell; K, keratin; KC, keratinocyte; KEAP1, Kelch-like erythroid cell-derived protein with cap‘n’collar homology-associated protein 1; NRF2, nuclear factor erythroid-2-related factor 2; Pso, psoriasis; ROS, reactive oxygen species; Th, helper T.

It is no doubt that the KEAP1-NRF2 system is dysregulated in the pathogenesis of AD and psoriasis (Figure 1). Because NRF2 plays a prominent role in redox homeostasis, cytoprotection, anti-inflammatory effects, and skin barrier function, considerable efforts have been made to develop NRF2-targeting drugs for AD and psoriasis. The most successful NRF2 activator to date is DMF, which has been demonstrated as a beneficial treatment option for psoriasis and MS, both clinically and experimentally. From a clinical perspective, NRF2 activators and inhibitors have both advantages and disadvantages. As reviewed here, NRF2 may play a dual role in the development of AD and psoriasis. Proper activation of NRF2 can ameliorate AD-like and psoriatic skin inflammation through its antioxidative and anti-inflammatory effects. Conversely, KEAP1-sensing of epidermal damage can lead to the activation of NRF2-mediated tissue-protective responses and exacerbate AD-like and psoriatic skin inflammation by inducing skin sensitization or KCs proliferation. Electrophiles, such as DMF and TBHQ, have shown that the treatment consequence may depend on the activation sites of NRF2. Further mechanistic approaches are necessary for the pharmaceutical applications of the KEAP1-NRF2 system.

## Abbreviations

AD	atopic dermatitis
AHR	aryl hydrocarbon receptor
Arp2/3	actin-related protein 2/3
Arpc4	actin-related protein 2/3 complex subunit 4
ATP	adenosine triphosphate
ca	constitutively active
CBT	*Chijabyukpi-tang*
CE	cornified envelope
CKS	Changkil saponins
CPD 6	chrysin-derivative
CPD 14	macakurzin C-derivative
CUL3	CULIN3
DAMP	damage-associated molecular pattern
DMBA	7,12-dimethylbenz(a)anthracene
DMF	dimethyl fumarate
DNCB	2,4-dinitrochlorobenzene
DNFB	1-fluoro-2,4-dinitrobenzene
DNTB	2,4-dinitrothiocyanobenzene
FAE	fumaric acid ester
FLG	filaggrin
GA	gallic acid
GAL	galangin
GSH	glutathione
HDM	house dust mite
HEK293	human embryonic kidney 293
HLA	human leukocyte antigen
HMGB1	high-mobility group box 1
IDMF	isosorbide dimethyl fumarate
IFN-γ	interferon-gamma
IgE	immunoglobulin E
IHC	immunohistochemistry
IL	interleukin
ILC	innate lymphoid cells
IMQ	imiquimod
JAK	Janus kinase
K	keratin
KC	keratinocyte
KEAP1	Kelch-like erythroid cell-derived protein with cap‘n’collar homology-associated protein 1
LOR	loricrin
LPS	lipopolysaccharide
MAPK	mitogen-activated protein kinase
MET	metformin
MMF	monomethyl fumarate
MQL	miquelianin
MS	multiple sclerosis
mTOR	mammalian target of rapamycin
NAC	*N*-acetylcysteine
NF-κB	nuclear factor kappa B
NHEK	normal human epidermal keratinocytes
NLRP3	nucleotide-binding oligomerization domain-like receptor pyrin domain-containing 3
NQO1	NAD(P)H quinone dehydrogenase 1
NRF2	nuclear factor erythroid-2-related factor 2
OMIM	Online Mendelian Inheritance in Man
OX	oxazolone
PAH	polycyclic aromatic hydrocarbon
POH	perillyl alcohol
PPI	protein–protein interaction
PsA	psoriatic arthritis
Pso	psoriasis
PsV	psoriasis vulgaris
RAGE	receptor for advanced glycation end products
ROS	reactive oxygen species
SC	stratum corneum
siRNA	small interfering RNA
SPRR2	small proline-rich protein 2
SST	*Soshiho-tang*
STAT	signal transducer and activator of transcription
TBHQ	*tert*-butylhydroquinone
TGN	tussilagonone
Tc	cytotoxic T
Th	helper T
TMA	trimellitic anhydride
TNCB	2,4,6-trinitro-1-chlorobenzene
TNF-α	tumor necrosis factor-alpha
TPA	12-O-tetradecanoylphorbol-13-acetate
Treg	regulatory T cell
UV	ultraviolet
VEGF	vascular endothelial growth factor
